# 5′ flanking region of *var* genes nucleate histone modification patterns linked to phenotypic inheritance of virulence traits in malaria parasites

**DOI:** 10.1111/j.1365-2958.2007.06009.x

**Published:** 2007-11-19

**Authors:** Jose Juan Lopez-Rubio, Alisson M Gontijo, Marta C Nunes, Neha Issar, Rosaura Hernandez Rivas, Artur Scherf

**Affiliations:** 1 Institut Pasteur-CNRS 25 rue du Dr Roux, 75724 Paris, France.; 2 Department of Molecular Biomedicine CINVESTAV, 7360, Mexico D.F., Mexico.

## Abstract

In the human malaria parasite *Plasmodium falciparum* antigenic variation facilitates long-term chronic infection of the host. This is achieved by sequential expression of a single member of the 60-member *var* family. Here we show that the 5′ flanking region nucleates epigenetic events strongly linked to the maintenance of mono-allelic *var* gene expression pattern during parasite proliferation. Tri- and dimethylation of histone H3 lysine 4 peak in the 5′ upstream region of transcribed *var* and during the poised state (non-transcribed phase of *var* genes during the 48 h asexual life cycle), ‘bookmarking’ this member for re-activation at the onset of the next cycle. Histone H3 lysine 9 trimethylation acts as an antagonist to lysine 4 methylation to establish stably silent *var* gene states along the 5′ flanking and coding region. Furthermore, we show that competition exists between H3K9 methylation and H3K9 acetylation in the 5′ flanking region and that these marks contribute epigenetically to repressing or activating *var* gene expression. Our work points to a pivotal role of the histone methyl mark writing and reading machinery in the phenotypic inheritance of virulence traits in the malaria parasite.

## Introduction

The persistence of the malaria parasite *Plasmodium falciparum* during the proliferation phase in red blood cells of its human host depends on the successive expression of variant molecules on the surface of the infected erythrocytes. This variation is mediated by the differential expression of a polymorphic parasite protein, *P. falciparum* erythrocyte membrane protein 1, which is encoded by ∼60 *var* genes (Kyes *et al*., 2001). Almost all *var* genes have promoter sequences of type *upsA*, *upsB* and *upsC*, with the exception of two highly conserved *var* genes, having *upsE* (*var2csa*) and *upsD* types (*var1csa*) (Kraemer *et al*., 2007). The process of *var* gene expression is apparently dependent on the strict control at the level of transcription initiation of full-length *var* genes, as has been shown using nuclear run-on experiments (Scherf *et al*., 1998; Kyes *et al*., 2007). *var* gene upstream regions are sufficient for controlling mutually exclusive transcription in the absence of the coding region (Voss *et al*., 2006) and repression was suggested to be modulated by *var* intron sequences (Dzikowski *et al*., 2006).

Growing evidence illustrates that the complex process of antigenic variation is orchestrated by epigenetic factors in the absence of any programmed DNA rearrangements. Although our understanding of *P. falciparum* epigenetic regulation is still in its infancy, it is increasingly evident that antigenic variation is controlled by a number of different factors. Apparently, the activation of a *var* gene is a multistep procedure including chromatin remodelling at *var* gene loci and relocation into a transcription competent region (Duraisingh *et al*., 2005; Freitas-Junior *et al*., 2005; Ralph *et al*., 2005a; Voss *et al*., 2006; Chookajorn *et al*., 2007). Telomeres appear to play a particular role in the control of antigenic variation, as this region promotes the nucleation of several putative silencing factors in the perinuclear region such as proteins homologous to silent information regulator (Sir) protein of the yeast *Saccharomyces cerevisiae* (Duraisingh *et al*., 2005; Freitas-Junior *et al*., 2005).

Switching in expression to an alternate *var* gene occurs at low frequency (Horrocks *et al*., 2004), indicating that cellular imprinting maintains an active *var* gene state during mitotic divisions and many parasite blood stage cycles.

**Fig. 1:**
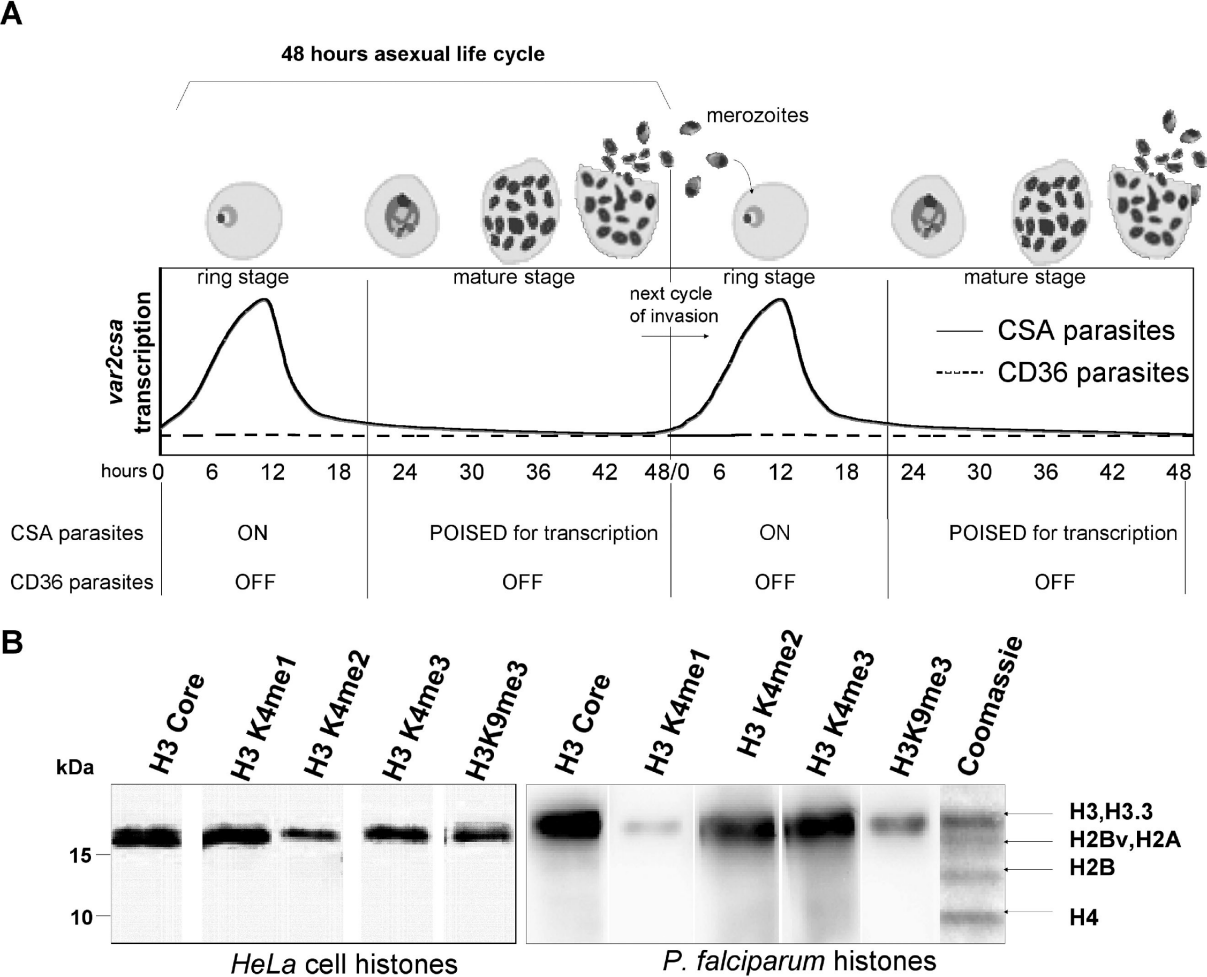
A. *P. falciparum* transcriptional *var* gene states during the 48 h blood stage cycle. Transcription of a single *var* gene starts about 4 h after the erythrocyte invasion by free parasite forms called merozoites and lasts for approximately 12–14 h (ring stage) (Kyes *et al*., 2000). For the remaining time of the 48 h cycle (trophozoite and schizont stage), which is mainly devoted to multiple rounds of parasite DNA replication and differentiation to merozoites, *var* gene transcription ends until free merozoites establish a new round of asexual blood stage cycle. Activation of a particular *var* member was achieved by selecting infected erythrocytes expressing a *var* gene able to bind to CSA, called *var*2*csa*. The two different states of transcription of an active *var* loci during the blood stage cycle is called here ‘ON’ for being transcribed and ‘POISED’ for being transiently silent but ready to get reactivated in the next cycle. Stable silencing (‘OFF’ state) of the *var2csa* gene is achieved by selecting parasites that express another *var* gene able to bind to CD36. B. Identification of histone H3 methyl marks in chromatin of asexual blood stage parasites. Equal amounts (∼8 μg per lane) of partially purified *P. falciparum* histones from mid to late trophozoites (30–34 h post invasion) or total HeLa cell extracts were separated by 15% SDS-PAGE. Various methyl modifications present on histone H3 were detected by immunoblotting with respective site-specific antibodies in equal dilutions.

Here we studied how the mono-allelic *var* expression pattern is transmitted from one parasite generation to the next. We investigated transcribed (ring stage), non-transcribed or poised state (schizont stage) and silent states of the same *var* gene in its natural chromosomal context (see Fig. 1A). We focused on post-translational modifications of histone N-termini, such as methylation and acetylation, which have been correlated with the functional organization of chromatin, gene expression and facultative heterochromatin (Lachner *et al*., 2004). Our results reveal that dynamic changes of histone H3 marks occur mainly in the 5′ flanking region of *var* genes indicating a prime role of this DNA element for epigenetic imprinting of ‘ON’ and ‘OFF’ states of antigenic variation genes.

## Results

### *P. falciparum* uses distinct histone H3 marks at lysine 4 and 9

To examine the presence in *P. falciparum* of histone modifications, we did protein blot analysis of acid-extracted histone preparations from asexual blood stage parasites (Fig. 1B). We found strong reactions with antibodies directed against histone H3 lysine 4 di- and trimethyl (H3K4me2/3) and much weaker reactions with H3K4 monomethyl (H3K4me1) and H3K9 trimethyl (H3K9me3). We observed strong reactivity with antibodies directed against H3K9 acetylation (H3K9ac) (data not shown) as has been previously reported (Miao *et al*., 2006). The same antibodies reacted with histone H3 of HeLa cell extract with similar intensity. These results are compatible with a differential use of methyl marks H3K4 and H3K9 in *P. falciparum*. For example, methylation of histone H3 lysine 9 is largely conserved from *Schizosaccharomyces pombe* to mammals. However, *Saccharomyces cerevisiae* chromatin does not carry this mark (Briggs *et al*., 2001). On the other hand, the difference seen in the antibody reactivity between HeLa cell and parasite histones may be caused by histone modifications in the neighbourhood of the studied marks.

To directly address the role of histone methylation and acetylation in antigenic variation, we performed analysis of the chromatin environment at the same *var* gene locus in an active and in a silent state in *P. falciparum* strain FCR3. We used a recently published receptor-panning assay, which allows the selection of a single *var* gene (*var2csa*) to be active or stably repressed (Viebig *et al*., 2005).

**Fig. 2:**
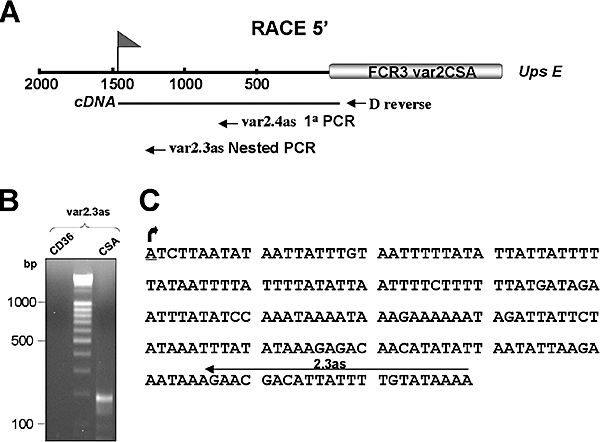
Determination of the transcription initiation site of the FCR3 *var2csa* gene. A. Schematic representation of the 5′*var2csa* region. The transcription initiation site of the *var2csa* gene is shown by a flag at position about −1475. The primers used for the RACE assays are indicated. B. The RACE products were analysed by gel electrophoresis on a 1.5% agarose gel. C. The sequence of the 200 bp DNA fragment obtained with the RACE 5′-assays with AUAP primer (Gibco) and nested *var2.3as* primer is shown.

**Fig. 3:**
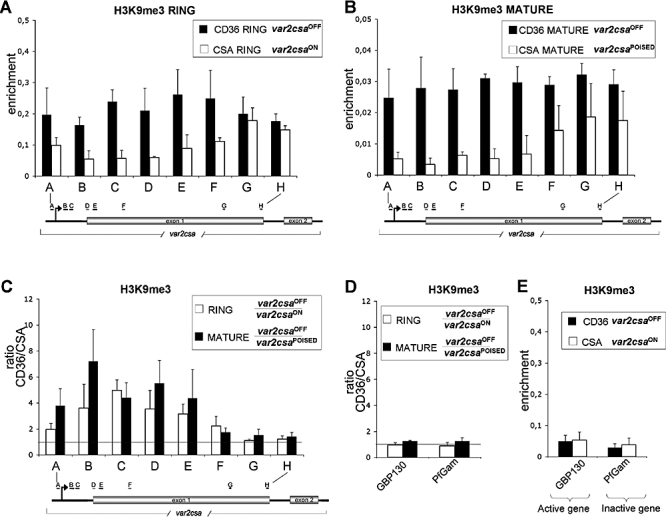
Trimethylated H3K9 levels at *var2csa*. A. Distribution of H3K9 trimethylation along the *var2csa* gene in CD36 (black bars) and CSA ring parasites (white bars). B. Distribution of H3K9 trimethylation along the *var2csa* gene in CD36 (black bars) and CSA mature parasites (white bars). C. Levels of trimethylated H3K9 (fold enrichment over the same positions in chromatin from CSA parasites) along the *var2csa* gene in CD36 parasites on ring stages (white bars) and mature stages (black bars). D. Levels of trimethylated H3K9 (fold enrichment over the chromatin from CSA parasites) at *GBP130* and *PfGam* genes in CD36 parasites on ring stages (white bars) and mature stages (black bars). E. Levels of H3K9 trimethylation at *GBP130* and *PfGam* genes in CD36 (black bars) and CSA ring parasites (white bars). qChIP values are given as DNA recovery (%input) normalized for the %input of total H3 for (A), (B) and (E). CD36/CSA ratio of the previous qChIP values is shown in (C) and (D). Regions are named as shown in Figure S1 and a simplified scheme is shown for reference. Results are the average of two or three independent experiments. Error bars denote standard deviation.

To map the histone modification patterns at the *var2csa* locus, we set up eight specific polymerase chain reactions (PCR) along a region of 10 kb comprising exon 1 (8 kb) and 2 kb upstream sequences (Fig. 3 and Fig. S1A). Examination of the upstream region by real-time PCR was possible for *var2csa* due to its uniqueness, unlike other *var* genes whose respective upstream regions fall into three main groups of conserved 5′ flanking regions (Kraemer *et al*., 2007). The transcriptional initiation site of *var2csa* was investigated by reverse transcriptase PCR (RT-PCR) and 5′-rapid amplification cDNA end (5′-RACE). For this purpose a first strand cDNA was synthesized from total RNA obtained from chondroitin sulphate A (CSA) and CD36 parasite population using a gene-specific primer (D reverse primer) (Fig. 2A and Fig. S1). Then a first PCR was performed using this cDNA as template and the gene-specific primer *var2.4as* followed by nested PCR using the gene-specific primer *var2.3as* and the first PCR as template generated a product around 200 bp (Fig. 2B). The PCR fragment was cloned and sequenced (Fig. 2C). Our results show that the initiation site is located approximately 1475 bp upstream of the ATG codon in FCR3. This result was confirmed using specific RT-PCR primers upstream and downstream of the initiation site and by Northern blot analysis (data not shown). It is noteworthy that a low abundance RNA was detected in the 5′ flanking region of *var2CSA* gene in parasites in which the gene is either repressed or active.

### H3 lysine 9 trimethylation is linked to stable repression of *var* genes

We investigated the role of H3K9 trimethylation in the control of antigenic variation. To this end we carried out quantitative Chromatin Immunoprecipitation (qChIP) analyses on *var2csa* loci in CSA parasites in which the *var2csa* is being transcribed (‘ON’ state termed here *var2csa*^ON^) or transiently repressed (‘POISED’ state termed here *var2csa*^POISED^) and in CD36 parasites in which the *var2csa* is stably repressed (‘OFF’ state termed here *var2csa*^OFF^) (Fig. 1A). All experiments were done in parallel with histone H3 core antibodies to normalize for potential differences in nucleosome density between active and silent genes. Our results show that H3K9 is highly methylated in the 5′ upstream region and extends into the coding region of exon 1 of the *var2csa*^OFF^ at ring stage (Fig. 3A and C). On the contrary, chromatin associated with the *var2csa*^ON^ gene was not marked by H3K9me3 at the 5′ flanking region and early coding region. The high levels of trimethylation of H3K9 associated with stably silenced *var2csa* at ring stage were maintained during entire asexual blood stage (Fig. 3B and C). This is consistent with the fact that *var2csa* is silenced throughout asexual stage in this population. In CSA parasites, the active *var2csa* gene is transcribed at high levels in ring stage. However, in mature parasites, *var2csa* transcription decreases 100-fold. But chromatin levels remain low for H3K9me3 marks, at least for the 5′ flanking region and early coding region. It is noteworthy that a slight increase of this mark is seen towards the *var* intron region of exon 1. Other types of silent *var* genes (*upsB and upsC*) are associated with high levels of methyl marks in H3K9 (Fig. S2) (Chookajorn *et al*., 2007). Thus, trimethyl H3K9 mark may be universally linked to *var* gene repression. This is distinct from PfSir2, which affects silencing of only a subgroup of telomeric *var* genes (*upsA* type) (Duraisingh *et al*., 2005).

An asexual blood stage gene, such as *GBP130*, is not enriched for this mark (Fig. 3D and E). We also studied a gametocyte transcribed gene *Pf14–0491* (http://www.plasmoDB.org) called *PfGam* here. The analysed position for silent *PfGam* was not associated with significant levels of H3K9 trimethyl marks.

### H3K4 tri- and dimethyl marks peak at 5′ flanking region of active *var* genes

**Fig. 4:**
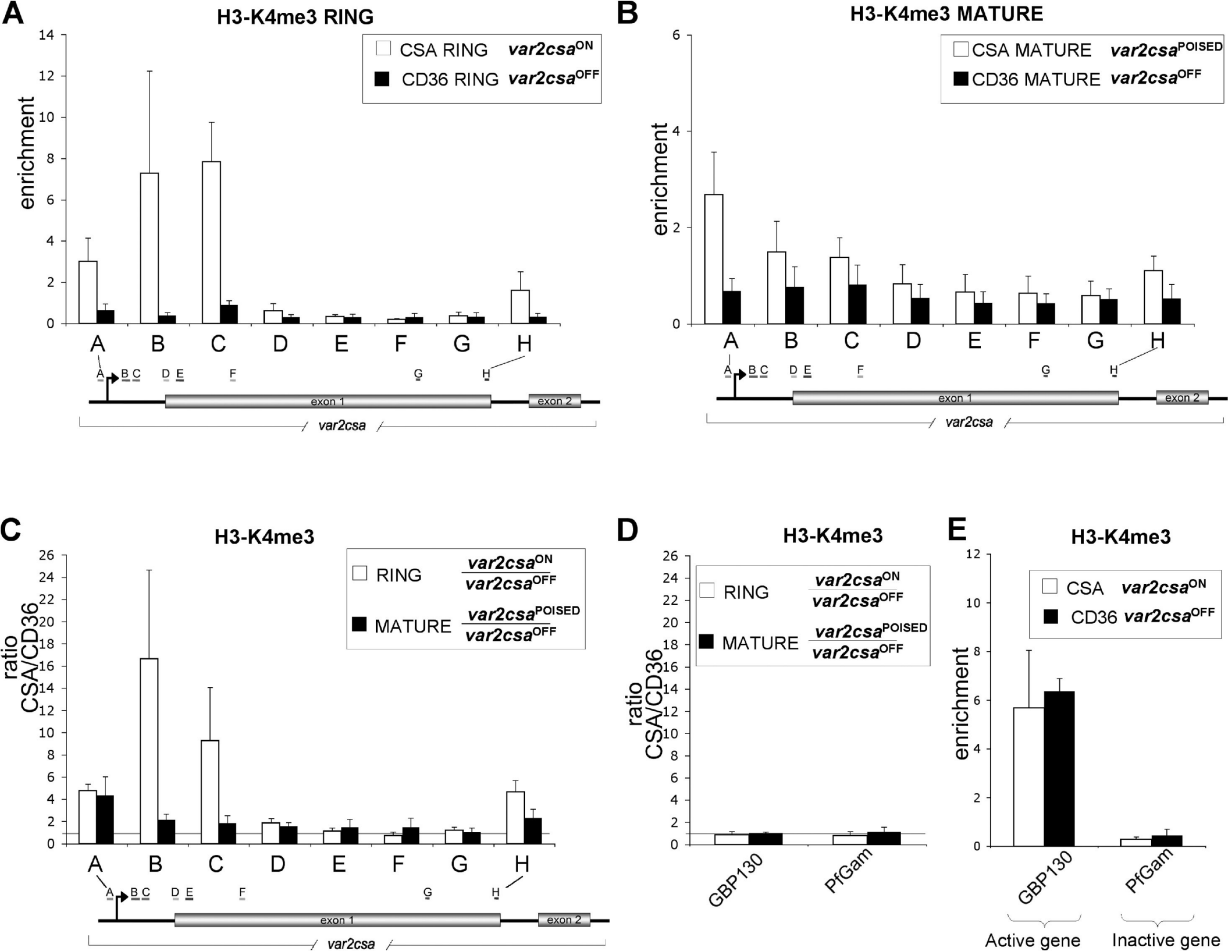
Trimethylated H3K4 levels at *var2csa*. A. Distribution of H3K4 trimethylation along the *var2csa* gene in CSA (white bars) and CD36 ring parasites (black bars). B. Distribution of H3K4 trimethylation along the *var2csa* gene in CSA (white bars) and CD36 mature parasites (black bars). C. Levels of trimethylated H3K4 (fold enrichment over the same positions in chromatin from CD36 parasites) along the *var2csa* gene in CSA parasites on ring stages (white bars) and mature stages (black bars). D. Levels of trimethylated H3K4 (fold enrichment over the chromatin from CD36 parasites) at *GBP130* and *PfGam* genes in CSA parasites on ring stages (white bars) and mature stages (black bars). E. Levels of H3K4 trimethylation at *GBP130* and *PfGam* genes in CSA (white bars) and in CD36 ring parasites (black bars). The data are plotted as for Fig. 3.

**Fig. 5:**
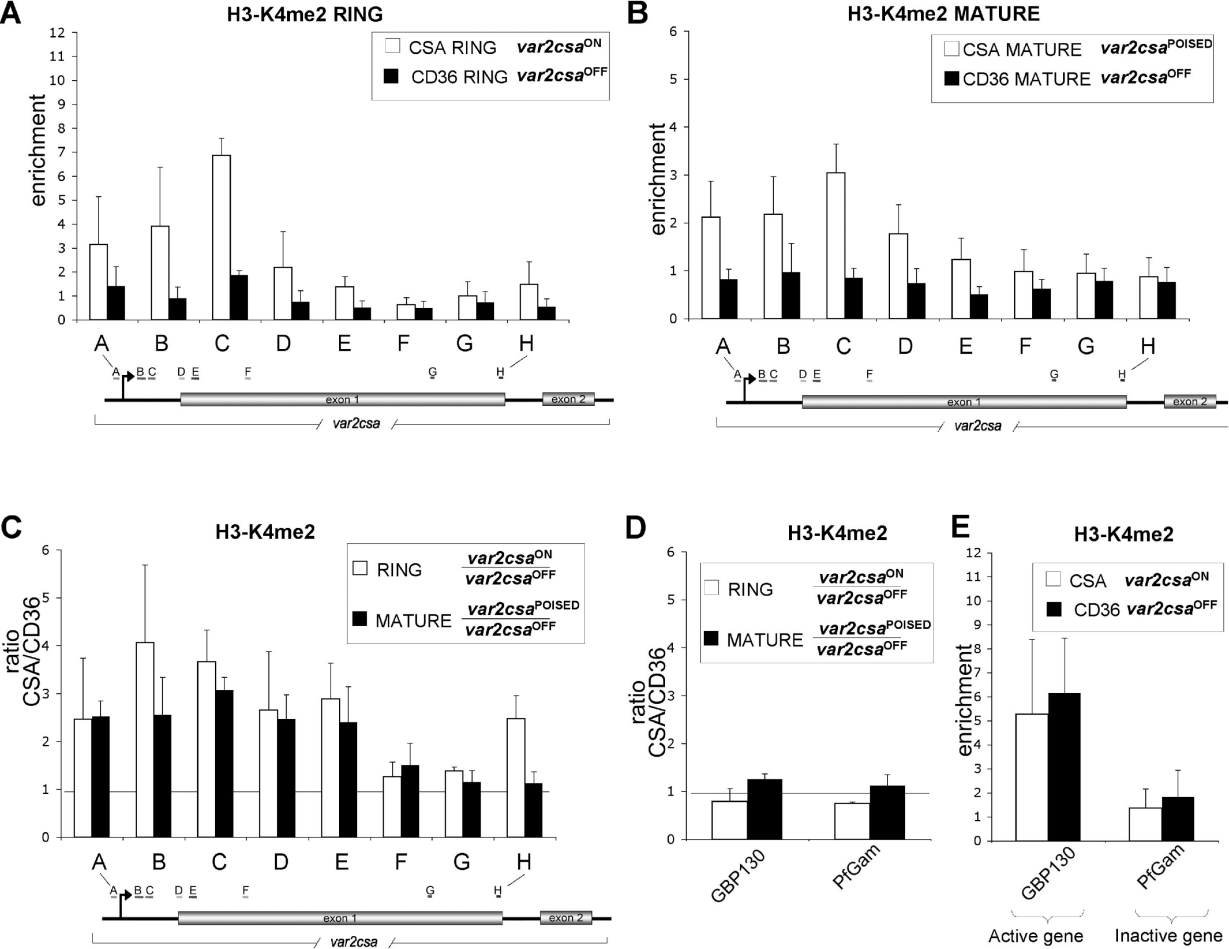
Dimethylated H3K4 levels at *var2csa*. A. Distribution of H3K4 dimethylation along the *var2csa* gene in CSA (white bars) and CD36 ring parasites (black bars). B. Distribution of H3K4 dimethylation along the *var2csa* gene in CSA (white bars) and CD36 mature parasites (black bars). C. Levels of dimethylated H3K4 (fold enrichment over the same positions in chromatin from CD36 parasites) along the *var2csa* gene in CSA parasites on ring stages (white bars) and mature stages (black bars). D. Levels of dimethylated H3K4 (fold enrichment over the chromatin from CD36 parasites) at *GBP130* and *PfGam* genes in CSA parasites on ring stages (white bars) and mature stages (black bars). E. Levels of H3K4 dimethylation at *GBP130* and *PfGam* genes in CSA (white bars) and in CD36 ring parasites (black bars). The data are plotted as for Fig. 3.

Particular histone methylation marks, such as H3K4 modifications, have been associated with transcriptional activity in other organisms. Trimethylated H3K4 is associated with active genes and dimethyl H3K4 correlates with a ‘permissive’ state of chromatin, in which genes are either active or potentially active (Schneider *et al*., 2004). We next investigated the role of these marks in *var* gene transcriptional control. ChIP analyses with antibodies against trimethylated H3K4 show that there is a prominent localized enrichment of trimethyl H3K4 at the 5′ flanking region of the *var2csa*^ON^ (Fig. 4A/C, A–C primers, white bars). There is also an enrichment of dimethylated H3K4 in the 5′ upstream region and early coding region of the *var2csa*^ON^ (Fig. 5A/C, A–E primers, white bars). Our results show that di- and trimethylated H3K4 marks differentiate active *var* members from stably repressed *var* gene members. To assess whether high levels of these modifications are a universal feature of active genes in *P. falciparum*, we analysed a single copy gene (*GBP130*) that is highly transcribed in asexual blood stage parasites (Figs 4D/E and 5D/E). The *GBP130* gene contains significant amounts of di- and trimethylated H3K4. The silent *PfGam* gene carries only low levels of these marks. Thus, similar marks are associated with the transcription of single copy genes in asexual blood stage parasites. Genome wide analysis of the H3K4 methyl mark using ChIP-on-chip could reveal, at a more global level, actively transcribed genes of *P. falciparum* blood stage parasites.

### H3K4 tri- and dimethyl marks are modified at poised *var* gene 5′ flanking region

While the active *var*2*csa* gene is transcribed only in early stages of erythrocyte infection in CSA parasites, the question arises how the parasite tags the *var*2*csa* locus during the non-transcribed trophozoite, schizont and merozoite stages in order to express the same gene in the next cycle. As H3K9 methylation marks do not appear to be used to transiently silence *var* genes in mature stages, we investigated the contribution of di- and trimethylation of H3K4 to bookmark the active *var* gene. We performed ChIP analyses of these modifications on chromatin where the *var2csa* is transiently repressed (*var2csa*^POISED^) and compared it to parasite chromatin preparations where the *var2csa* is stably repressed (*var2csa*^OFF^). These results show that levels of tri- and in particular dimethylation of H3K4 are higher for *var2csa*^POISED^ compared with *var2csa*^OFF^ (Figs 4B/C and 5B/C). The enrichment of trimethylated H3K4 on *var2csa*^POISED^ gene is notably lower than during the transcribed state (Fig. 4C). For the dimethylated H3K4, the enrichment at the 5′ flanking region and early coding region associated with *var2csa* in its ON state is maintained when the *var2csa* is not being transcribed but poised for transcription (Fig. 5C, A–E primers). This indicates that these marks are not only involved in *var* transcription but apparently are required to maintain the poised state and avoid the reorganisation by repressive chromatin of this *var* into a stable silent state.

### Antagonist distribution between H3K9me3 and H3K9ac at active and silent *var2CSA*

**Fig. 6:**
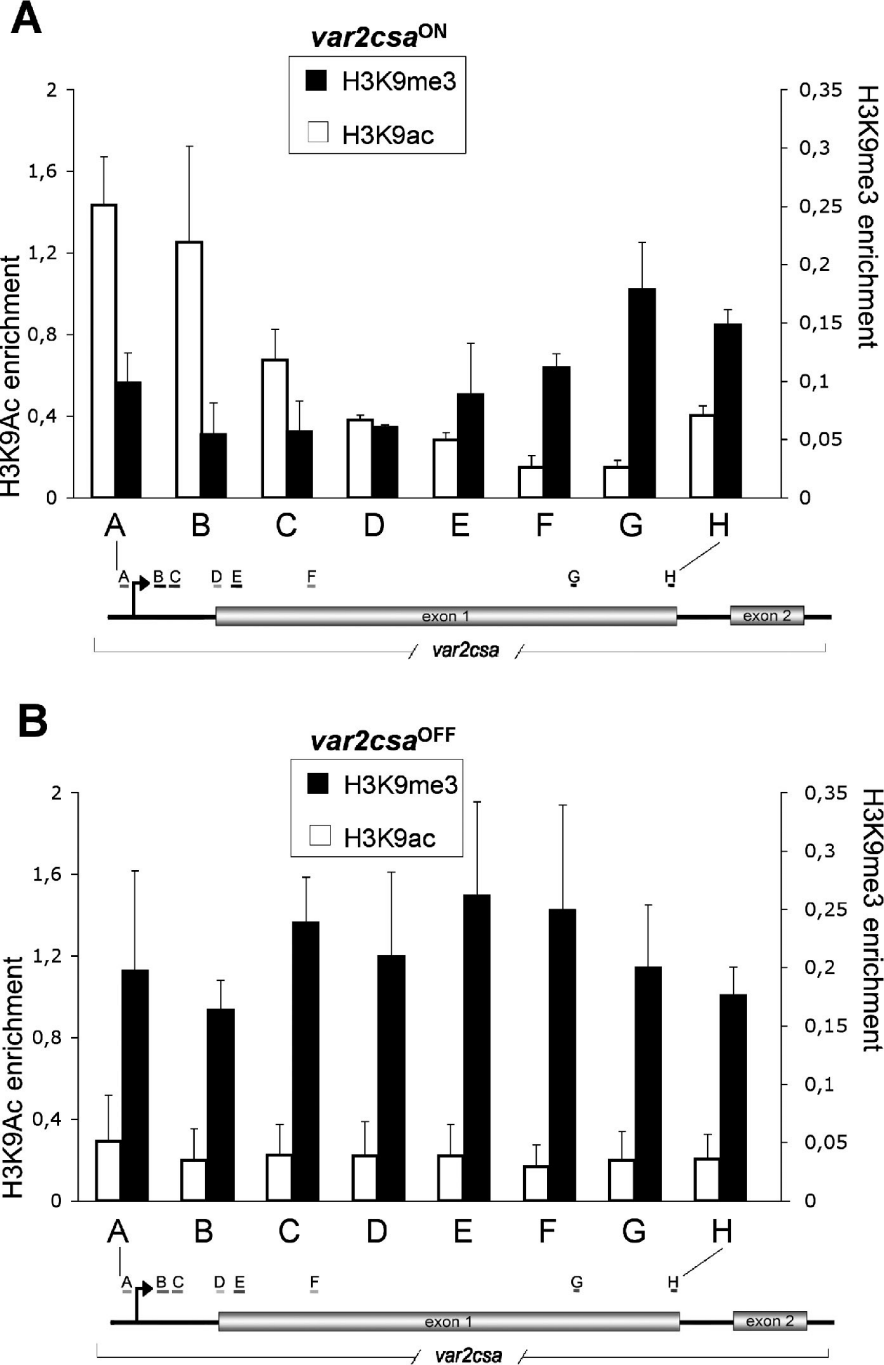
Acetylated and methylated H3K9 levels at *var2csa*. A. Distribution of H3K9ac (white bars) and H3K9me3 (black bars) along the active *var2csa* gene in FCR3 CSA ring stage parasites. B. Distribution of H3K9ac (white bars) and H3K9me3 (black bars) along the silent *var2csa* gene in FCR3 CD36 ring stage parasites. The data are plotted as for Fig. 3. Note that the H3K9ac antibody gives stronger signals and that the *y*-axes are different for the two antibodies.

As the active *var2csa* gene is deprived of H3K9me3 at the 5′ upstream region (Fig. 3) and H3K9ac can be a mark of active housekeeping genes in *P. falciparum* (Cui *et al*., 2007), we investigated weather the methylation mark of H3K9 would be replaced by acetylation in the active *var* gene. To test this hypothesis, we analysed H3K9ac across the *var2csa* gene and compared this with H3K9me3 in its active and silent state (Fig. 6). H3K9ac was specifically enriched at the 5′ flanking regions of the active *var* gene, showing an opposite distribution of that observed for H3K9me3. Thus, both histone H3 acetylation and methylation marks define active *var* states yet in distinct N-terminal lysine positions.

## Discussion

**Fig. 7:**
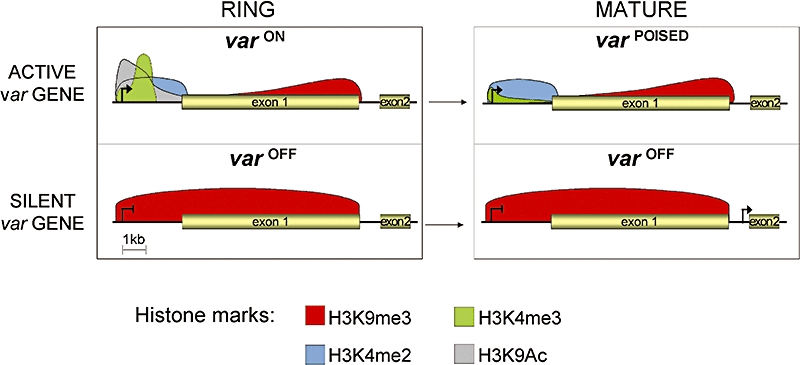
Schematic presentation of histone H3 marks linked to silent or active *var* genes. Histone modifications of the 5′ flanking region and exon 1 of an active (*var2csa*^ON^ or *var2csa*^POISED^) and silent *var* gene loci (*var2csa*^OFF^) are shown. H3K9me3 is dynamically removed in the surrounding area of the transcription start site upon gene activation and is restored upon repression. Histone H3 marks at lysine 4 and 9 linked to active *var* genes peak in chromatin associated with 5′ flanking *var* region, supporting further the concept for a key role of this DNA element in the control of mono-allelic expression of *var* genes (Dzikowski *et al*., 2006; Voss *et al*., 2006). Exon 2 is transcribed via a promoter-like sequence within the *var* intron sequence element in silent *var* genes and has been linked to *var* gene silencing (Dzikowski *et al*., 2006; 2007). Chromatin associated with *var* intron and exon 2 has not been analysed due to the sequence homology of these regions with most members of the *var* gene family.

In this work we have investigated histone marks at different positions in the coding and 5′ flanking region of the *var2csa* gene. In the repressed state, the large Exon 1 and the 5′ upstream region are associated with high levels of methyl marks in lysine 9 of histone H3. Based on this observation it was difficult to spot a critical DNA region that could seed the repressive methyl mark. The study of the active *var2CSA* gene, however, revealed that the 5′ flanking region undergoes striking histone mark changes, with high peaks of methyl marks in lysine 4 and acetylation marks in lysine 9 (see model in Fig. 7). This observation supports the earlier finding by Voss *et al*. (2006) showing that a *var* 5′ upstream region of ∼2 kb is sufficient to control mono-allelic expression of *var* genes and that no expression of *var* genes are needed to achieve mutually exclusive expression (Dzikowski *et al*., 2006; Voss *et al*., 2006). Dynamic histone mark changes are not limited to the 5′ flanking region. We observed ‘active’ marks, at the border of exon 1 with the intron, although at a lower level compared with 5′ flanking region, in transcribed and poised *var2csa* (Figs 4–6, position H). This may reflect transcriptional activity in the intron region of active *var* genes. Indeed, we have recently shown that *var2csa* antisense RNA is produced and may originate from a cryptic promoter in the intron region (Ralph *et al*., 2005b).

Our data raise the possibility that the repressive H3K9me3 mark may contribute mainly to the differential transcription of members of other subtelomeric antigen gene families, such as *rif* (Fernandez *et al*., 1999) or the reticulocyte homology binding-like protein family (PfRh) (Stubbs *et al*., 2005) expressed at the surface of merozoites. A recent study, however, presented evidence that trimethyl H3K9 was enriched in 5′ flanking regions of non-subtelomeric genes of *P. falciparum* such as MAL7P1.38 and DNA polymerase (Cui *et al*., 2007), suggesting a more universal use of this mark in gene silencing. However, in this study, no trimethyl H3K9 was observed in the coding region of the analysed genes. By contrast the enrichment is present along the whole gene for silent *var* genes, indicating that the enrichment of H3K9me3 in subtelomeric coding regions is linked to the particular heterochromatin state of these chromosome regions.

A previous study demonstrated that *var* gene activity correlates with histone H4 lysine acetylation in the 5′ upstream region whereas *var* gene silencing correlated with low levels of acetylation and the presence of PfSir2, a histone deacetylase. Here we extended the analysis to histone H3 acetylation. We observed that lysine 9 has a key position in *var* gene regulation. We found that 5′ flanking *var* regions are either highly acetylated in the active state or vastly trimethylated in the silent state, indicating an antagonistic distribution of distinct histone marks at active *var* genes. It further demonstrates that *var* gene activation is a complex multistep procedure at the histone mark level, which is part of a cascade of events that leads to *var* gene expression. This implies that a number of different histone-modifying enzymes (acetylases, methylases, deacetylases and demethylases) need to be recruited to this site in order to co-ordinate expression of *var* genes.

Specific histone methyl mark binding chromodomain proteins, such as HP1, may translate this information into stably repressed chromatin (Lachner *et al*., 2001). Several candidate proteins carrying a chromodomain have been identified in the genome of *P. falciparum*. We have initiated experiments to elucidate their role in *var* gene silencing.

A recent study analysed histone marks associated with silent *var* genes during ring stage (Chookajorn *et al*., 2007). These authors investigated a *var* gene promoter controlling a selectable marker (blasticidin-S-deaminase gene, BSD). An active *var* promoter state was artificially mimicked by selecting blasticidin-resistant parasites. As the analysis of the 5′ upstream region is compromised by the sequence homology with numerous *var* genes, the authors analysed a single region in the BSD gene or silent *var* genes. Based on their results, they concluded that H3K9me3, but not H3K9me2 or monomethylation, correlates with repression of *var* genes. However, our data clearly show that most changes in the histone methyl and acetyl marks occur at the 5′ flanking region of *var* genes, indicating that investigating coding regions gives only a limited insight into critical DNA elements involved in epigenetic regulation of antigenic variation. With regard to H3K9me3, the study of Chookajorn *et al*. supports our finding, because this mark spreads from the 5′ flanking region into the coding region of *var* genes.

The question arises how an active *var* gene state is maintained during many cell generations. We hypothesize that cellular memory may be acquired by inheriting a *var* locus enriched with H3K4me at the 5′ flanking region. Active transcription restarts in the same ‘marked’*var* gene, possibly by raising histone trimethyl marks again to levels needed for transcription. Dynamic changes of H3 lysine 4 methylation marks in the 5′ flanking region have been observed in higher eukaryotes and yeast and correlated with transcriptional activation or permissive state of genes (Santos-Rosa *et al*., 2002; Schneider *et al*., 2004).

Our findings may be relevant for a number of other protozoan pathogens that use antigenic variation and in the mammalian neuronal cells that express a single member of the large odorant receptor gene family (Borst, 2002). For example, in African *trypanosomes* the mutually exclusive transcription of a *vsg* gene is mediated by a particular subnuclear compartment called the ‘Expression Site Body’ (Navarro and Gull, 2001). In mouse olfactory sensory neurones, this is achieved by a particular inter-chromosomal interaction of a single enhancer with an odorant receptor gene promoter (Lomvardas *et al*., 2006). These specific subnuclear interactions are necessary to preserve mono-allelic expression. However, DNA replication and cellular divisions probably temporarily disconnect their association. H3K4 histone marks may contribute to stably maintaining an active gene poised during mitosis, avoiding inadequate switches in expression between members of these gene families.

In conclusion, our findings have important implications for the processes that promote immune escape and pathogenesis in human malaria parasite. The presented data overcome a conceptual gap in our understanding of antigenic variation, namely, how *P. falciparum* generates reiterated *var* gene expression patterns through multiple blood stage cycles. Our data show that H3K4 lysine and H3K9 lysine methylation/acetylation marks at the flanking region of *var* genes are involved in setting up distinct *var* gene states and this in a strictly mutually exclusive manner. Future research will focus on the molecular machinery that can read these marks at particular DNA regions of the 5′ upstream region and translate this information into co-ordinated gene expression pattern of *var* genes.

## Experimental procedures

### *P. falciparum* cultures and panning

*P. falciparum* blood stage parasites from FCR3 strain and panning assays for selection of FCR3 parasites that transcribed *var* genes associated with CD36 and CSA binding were performed according to Scherf *et al*. (1998).

### Purification of *P. falciparum* histones and Western blot analysis

Parasite histones were purified by modification of a previously described procedure (Longhurst and Holder, 1997). Precipitated histone proteins were analysed by 15% SDS-PAGE and transferred onto nitrocellulose membranes. Membranes were probed with commercially available antibodies against core histone H3 (Abcam, ab1791), and antibodies against mono-, di-, trimethylation at histone H3K4 (Abcam, ab8895, ab7766, ab8580 respectively), trimethylation at histone H3K9 and acetylation at H3K9 (Upstate, 07-442 and 07-352 respectively). Secondary antibodies conjugated to horseradish peroxidase (Pierce) were developed with SuperSignal West Pico Chemiluminescent Substrate (Pierce) according to the manufacturer's instructions.

### Chromatin immunoprecipitation and quantitative real-time PCR

The chromatin immunoprecipitation assay was performed as described previously (Freitas-Junior *et al*., 2005). Around 15 μg of chromatin per immunoprecipitation was used and immunoprecipitation was performed at 4°C overnight with a 1:100 dilution of anti-H3K9me3, anti-H3K9ac and anti-H3 core and a 1:150 dilution of anti-H3K4me2 and anti-H3K4me3. Immunoprecipitated DNA was analysed by Real-time qPCR (Realplex4 EpgradientS thermal cycler from Eppendorf) using SYBR Green and standard settings (Eppendorf). PCR was performed in duplicates and serial dilutions of purified input DNA were measured together with the immunoprecipitated DNA samples. This allowed us to calculate the amount of target sequence in immunoprecipitated chromatin relative to the amount of target sequence in input (%input). This value was normalized for the %input of signals obtained with histone 3 core antibodies. Melting-curve analysis was done at the end of each program to assess specificity of the amplification; specificity was further determined by showing the presence of a single band on ethidium bromide-stained gel. The sequences of primers used are shown in Fig. S2B. The presented data are the average of two or three independent immunoprecipitations from different parasite extracts. The ChIP recovery of anti-H3 core antibodies was significantly higher in schizont stage than in ring stage (seven to nine times) in both CSA and CD36 parasites. Although the cause for this observation is not clear to us it may be due to differential modifications during blood stage development. As a consequence the ChIP enrichment in schizont stage after correction with the anti-H3 core antibodies is reduced. However, presenting the results as a ratio between CD36/CSA eliminates this difference (Fig. 3C). We did not find major differences for histone H3 density between transcribed and non-transcribed *var2csa* at ring stage and between poised and silent *var2csa* at schizont stage.

## Supplementary material

This material is available as part of the online article from:

http://www.blackwell-synergy.com/doi/abs/10.1111/j.1365-2958.2007.06009.x

(This link will take you to the article abstract).
