# Supplementary material for: 5′ flanking region of var genes nucleate histone modification patterns linked to phenotypic inheritance of virulence traits in malaria parasites
Source: Mol Microbiol. 2007 Nov 19;66(6):1296–305. doi: 10.1111/j.1365-2958.2007.06009.x (PMC2228885; doi:10.1111/j.1365-2958.2007.06009.x)

Fig. S1

A

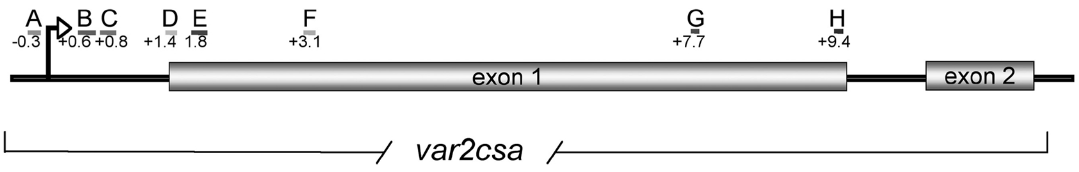

B

List of PCR primers used in this study

Name	Forward primer	Reverse primer
A	TGCTTCATAAATAAAACATGCAA	TTCTTGTTTTTCTAAAATACCTCTCTT
B	CACGACATTAACAATACATGCAGA	TGTCATTGCATTCACACACAA
C	ATGGTGATGTATGTGTTTATGG	TTATCGTGTAAGGGTATCTGAC
D	GGACAAAATGGATAGTACAAGCAC	TTTAAGTAATCTCCATCACCTCCA
E	CTCCGAGAAGAGAAAGATTATG	AAAGCATTACAAACATTGGAAC
F	AAATGCGACAAATGTAAATCTG	GGGGTAAACCTATGGTGTTAGC
G	AGCCCAATCGGAAGGTAAGT	TTCATAGCTTCTAGCGCCTT
H	TGCCCAAAACCTTTACCATCCC	ACCCTAACGCCAACGCAATACC
GBP130	TGTTTTCCCTGAACCTTTTTTC	AACAAATGATGCTAAGGTAAAATATATG
PfGam	TTTTCAATTTACTCCTGTACTTTATACCA	TTCTAGTGTCATACCTTTAAATTTTCC
IT4 *var19*	AAATGGTAAGGCAGTTGCGTTG	TGGCGTTCCTATGTCAAACCTG
IT4 *var44*	TGATGCCAATGATGCTAAGG	CCCCAACAAAAATTGATGACC
2.3as	TTTTATACAAAATAATGTCGTTCT	
2.4as	ATCACCATAATTAATATGAGAATC	

Fig. S2

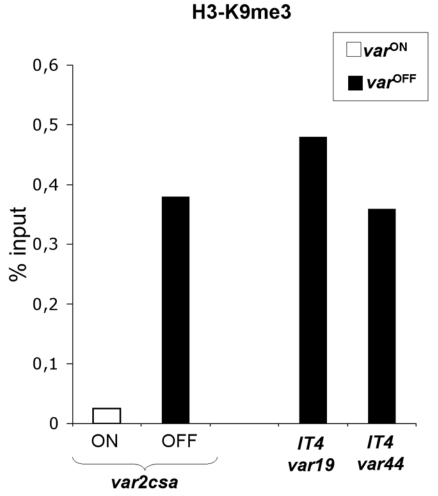

Supplement: Supplementary file 1 [file mmi0066-1296-SD1.pdf]
